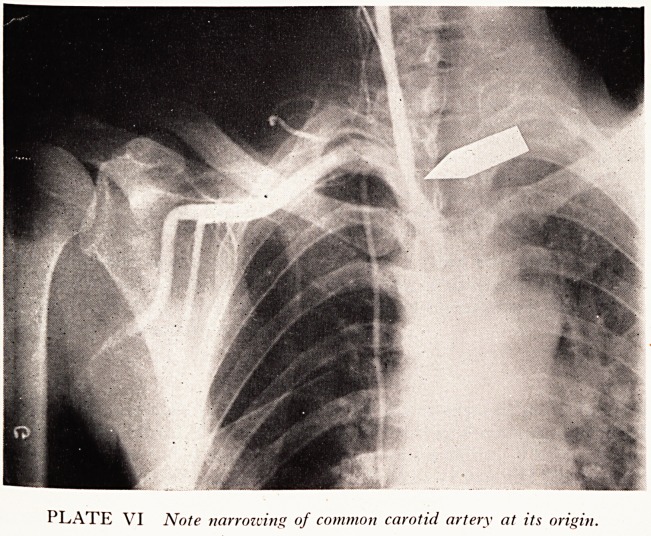# Disease of Major Cerebral Vessels

**Published:** 1962-01

**Authors:** J. L. G. Thomson

**Affiliations:** Radiologist, Frenchay Hospital, Bristol


### DISEASE OF MAJOR CEREBRAL VESSELS

BY

J. L. G. THOMSON

*Radiologist, Frenchay Hospital, Bristol*

In 1927 Egaz Moniz, Professor of Medicine at Lisbon University, presented a paper to the Neurological Society of Paris, oulining his discovery of cerebral angiography. He had used strontium bromide and then sodium iodide, the injection being made following a cut-down operation on the artery in the neck. In 1937 he recorded the first case of carotid artery occlusion to be demonstrated radiologically. Since then numerous papers have been written on this subject and at a recent count Luessenhop (1959) found 486 cases in the literature.

Nowadays, we regard cerebral angiography as a fairly simple and straightforward procedure. It is performed by percutaneous puncture of the artery in the neck, adults being mildly sedated and children receiving a general anaesthetic. Rapid injection of an organic iodine compound is made, "Hypaque" being the choice at the present time, and several films are taken in quick succession from the side of the head, and after a further injection, from the front. Further views may then be required depending on the nature of the case, but on an average the whole examination takes about half an hour, and in terms of discomfort compares favourably with the drilling of a tooth. It is a most helpful diagnostic aid, and an estimate of its value can be gained from the fact that 650 patients underwent this procedure at Frenchay Hospital last year, and none of them suffered any permanent ill effects. About 5–10 per cent of patients, notably those with cerebral tumours, may deteriorate a little for the ensuing few hours but they then return to their former state.

In the Neurological X-ray Department patients are investigated from the medical and surgical wards of this and other hospitals in the locality, and in the 3,000-odd patients thus seen in the last 5 years, we have encountered ninety-one cases of partial or complete obstruction in the carotid or middle cerebral arteries which was considered to be the cause of the patients' symptoms. We have also carried out several vertebral angiograms to detect stenosis at the origin of the vertebral artery or partial block in the basilar artery or its branches, but although we have suspected abnormality in one or two cases no proof has been forthcoming, and these cases are therefore not included in the series.

In contemporary medical literature, considerable interest is being focused on the follow-up of "strokes", so as to obtain a base line from which to assess different forms of treatment. Marshall and Shaw in 1951 followed up a series of patients who had left hospital with a diagnosis of cerebro-vascular disease, and they found that over half of the patients with unilateral hemisphere lesions were dead in the course of the next few years. Lindgren (1958) followed up a series of patients with occlusion of the internal carotid or the middle cerebral arteries, and found that a third of them were dead within the ensuing two years. As this seemed to be the only article in the literature dealing with the follow-up of this particular group of "strokes", it was felt worth while to analyse our ninety-one cases more closely and assess their prognosis.

### MATERIAL

Table I shows the anatomical breakdown of these ninety-one cases, and it will be noted that the majority of the lesions occurred at the origin of the internal carotid artery in the neck. There were a few in the siphon, which is the part of the internal caroti[illegible] artery alongside the sella turcica; and a smaller number occurred in between these tw[illegible]

TABLE I

Nature and Site of Obstruction

| | | |
|---|---|---|
| Common carotid artery | | |
| | *Partial obstr.* | 1 |
| | *Complete obstr.* | 1 |
| Origin of internal carotid artery | | |
| | *Partial obstr.* | 19 |
| | *Complete obstr.* | 34 |
| Carotid siphon | | |
| | *Partial obstr.* | 8 |
| | *Complete obstr.* | 8 |
| Mid-portion of internal carotid artery | | |
| | *Partial obstr.* | 3 |
| | *Complete obstr.* | 2 |
| Middle cerebral artery | | |
| | *Partial obstr.* | 3 |
| | *Complete obstr.* | 12 |
| | TOTAL | 91 |

extremities, and in the common carotid artery. The lesions in the middle cerebr[illegible] artery were mostly at or close to its origin at the base of the brain. The remainder [illegible] this article mainly illustrates cases from some of these groups with particular emphas[illegible] on the follow-up.

In the ninety-one cases, males outnumbered females by two to one, almost all ag[illegible] being represented, from 8 years of age to 81, the average being 53 years. It did see[illegible] apparent from these figures that middle-aged males were the commonest victims.

As regards the onset, about one-third of the patients suffered a sudden or dramat[illegible] onset of hemiplegia or hemiparesis, although at arteriography only two-thirds of the[illegible] showed a complete vessel block, the others showing a partial block only. On the oth[illegible] hand, about one half of the ninety-one patients had a history of as long as one mon[illegible] or more, either of a slowly progressive unilateral weakness or of the so-called stutterin[illegible] type—the cerebral intermittent claudication of Ramsay Hunt. This was often of tin[illegible] lings, numbness or weakness of a few fingers or a hand lasting a few minutes, occasio[illegible] ally with temporary blindness in the opposite eye, attacks which progressed in fr[illegible] quency and severity until the changes often became permanent. A few of the ninety-o[illegible] patients had had symptoms for as long as one year or even more. From the variabili[illegible] of the clinical picture, it is clear that other pathological conditions, particularly perha[illegible] cerebral tumour, may present with very similar and even indistinguishable case histo[illegible] ies, and it is in differentiating these underlying lesions that arteriography so often pla[illegible] a useful part. To look at the subject the other way round, it might be mentioned th[illegible] arteriographic analysis of a series of so-called "strokes", both major and minor, wou[illegible] show that about one-quarter of them were due to lesions in the carotid or midd[illegible]

cerebral arteries, that about one-half of them showed no demonstrable lesion (these probably being the ones in which the tiny vessels to the internal capsule were involved), and that the rest showed one of many lesions such as haematoma with or without an angioma or aneurism, or a tumour, either glioma or meningioma, amongst others.

A plaque of atheroma is quite a common finding at the origin of the internal carotid artery. Hultquist of Jena (1941) performed 3,500 consecutive autopsies thoroughly, and found atheromatous plaques at this site in ninety-one cases (2·6 per cent). Northcroft and Morgan (1944) examined the artery in thirty consecutive autopsies on military men who had died from other causes, and found a variable degree of atheroma at this site in almost every case. At arteriography for other conditions, e.g. for investigation for possible subdural haematoma following head injury, an incidental plaque-like filling defect may be encountered at the origin of the internal catorid artery, and this we regard as of no clinical significance; but if the vessel narrowing were more pronounced, there would come a stage in the appropriate case when clinical symptoms would ensue. From this point of view it is worth recalling Poiseuille's law, which states that when pressure and viscosity are constant, the rate of flow in a narrow tube is inversely proportional to the length of the tube and directly proportional to the fourth power of the radius. In the human body, two main modifying factors would be operative, firstly the blood pressure, and secondly the degree of collateral supply available, this for the brain being dependent mainly on the circle of Willis. Obviously with well developed communicating arteries the circle of Willis would be able to compensate for a poor or absent blood supply from one carotid artery, but if these vessels were small or absent, then it might be impossible to shunt blood across from one side of the brain to the other. Doubtless the variability in the severity of symptoms from minimal complaints such as temporary blindness in one eye to dense hemiplegia is largely explained on this basis.

### INTERNAL CAROTID ARTERY OBSTRUCTIONS

The situation of partial vessel obstruction is illustrated in the following case:

*Case History*

A parson from Gloucester aged 60 years had been having attacks of transient hemiparesis for several months, the right arm shaking in an attack, with later development of dysphasia which proved an embarrassment to him on occasions in the pulpit. His left eye had recently been blind for two days. The arteriogram (Plate I) showed gross narrowing of the internal carotid artery in the neck. Mr. R. E. Horton operated on the patient as an emergency, inserting a by-pass arterial graft. He made a good recovery and returned to light parish duties for a while. It is now three years later and it is understood that he has recently retired to Devon in quite good physical health.

A further similar case is worth quoting as we have pre-operative and post-operative films of this carotid artery (Plate II).

*Case History*

A scrap metal dealer aged 52 years had complained for 6 months of tinglings in his right fingers followed by weakness and numbness of the hand, with the result that he was initially referred to the hospital orthopaedic out-patient department. Later, he had an attack of blindness in his left eye lasting five minutes. A bruit was then heard in his neck over the left carotid artery and arteriography was performed, demonstrating marked narrowing and irregularity of the arterial lumen as shown in the left-hand picture of Plate II. Professor Milnes Walker carried out an endarterectomy on this artery with extremely good results, in that the power and sensation rapidly returned to the hand following operation. The post-operative arteriogram in the right-hand picture of Plate II shows that the lumen of the vessel has been much enlarged. He is still very well two years later, being maintained on anticoagulant therapy.

Further similar cases could be quoted. However, it might be worth noting that in one patient, a lady of 43 years with a six months' history, a saddle embolus was found astride the bifurcation of the common carotid artery, so that even in this small serie atheroma is not always the cause of the partial obstruction.

From the point of view of the follow-up of the nineteen patients in this group, te were treated medically, and of these two are now back at work 2 to 7 months after diag nosis, three remain hemiparetic, and five are dead 3 weeks to 1 year later. Of the nin cases treated surgically by various surgeons, four are well and back at work 15 month to 3 years later, two remain hemiparetic, and three died within a few days of operatio

There were thirty-four patients with complete obstruction in the internal caroti artery at its origin in the neck and the following case is of special interest as occlusio may have been precipitated by dehydration.

*Case History*

A man of 30 years of age suffered from "gastric 'flu", and whilst recovering from this he had severe headache one evening and awoke next morning to find his left arm paralysed. Arterio graphy (Plate III) shows that the internal carotid artery is completely occluded close to it origin in the neck.

Of these thirty-four cases, eleven are now well and back at work 5 months to 3 year after diagnosis, fourteen remain hemiparetic, and five died within a few weeks. Surger was performed in three cases in an attempt to re-establish blood flow, but as has bee found at other centres the results are not good, presumably because the brain is alread infarcted and the damage is beyond any quick recall. Of these three patients, two die very shortly afterwards and the fate of the third is unknown. One other patient coul not be traced.

### SIPHON OBSTRUCTIONS

Of the eight patients with a partial obstruction in the carotid siphon, the followin case is an interesting example.

*Case History*

An American truck driver aged 47 years was over in Bristol visiting his in-laws. He consulte Dr. A. M. G. Campbell because of increasing weakness in his left arm, developing slowly ove the preceding four months. It was suspected that he had a cerebral tumour and arteriograph was performed. This showed no evidence of tumour, but instead there could be seen marke irregularity and narrowing of the internal carotid artery in its course alongside the sella turcic (Plate IV). It was feared that the partial obstruction would in time become complete, and th a hemiplegia would develop. Anticoagulants were therefore commenced but he develope haematuria after three weeks and they were therefore stopped. Contact with the patient wa then lost for a time as he went back to the U.S.A., but a recent inquiry 2 years later has show that he did in fact deteriorate a little over the subsequent few weeks but then gradually improve so that for the last 12 months he has been fit enough to resume his occupation.

Interestingly enough, this satisfactory outcome has occurred in four out of the eigh patients over a period of 6 months to 2 years, and a fifth patient has returned to wor only to die after 2 years from a myocardial infarction. The other three patients all ap pear to be improving 8 months to 1 year after diagnosis. It does appear that in thi group, with a slow onset of symptoms and a partially obstructed artery, adequate col lateral supply has developed over the course of time. We have been unable to obtai follow-up arteriograms on any of these patients to see whether the artery is still partial ly or is now completely obstructed.

Of the eight patients with a complete obstruction in the siphon, one is particularl worth recording.

*Case History*

A boy aged 12 years fell off his bicycle and hit his head on the road. He was unconsciou for five minutes and was then found to be hemiplegic. Plain X-ray examination of his hea showed several fracture lines in the vault indicating the severity of the blow, and arteriograph

PLATE I *Note almost complete obstruction of internal carotid artery in the neck*

PLATE II *Note partial obstruction of internal carotid artery before (left) and after (right) operation.*

PLATE III

*Note complete obstruction of i[illegible]*

*nal carotid artery in the ne[illegible]*

PLATE IV

*Note marked irregularity and narrowing of carotid siphon.*

PLATE V *Complete obstruction of middle cerebral artery. Note the stump of the artery in the right hand picture.*

PLATE VI *Note narrowing of common carotid artery at its origin.*

showed complete obstruction of the siphon just beyond the origin of the posterior communicating artery. Arteriography was then performed on the other carotid artery and this showed an excellent cross-circulation, indicating that a good blood supply was being provided to both cerebral hemispheres from this one carotid artery. The obstruction in the first artery examined would seem to be the result of a "shearing" strain on the vessel, shortly beyond its emergence from the fairly rigid dura around the cavernous sinus. This is a rare but recognized lesion occurring in head injury cases. The boy returned to school in fairly good shape two months later.

Follow-up of this group shows that four of them are now well 1 to 5 years after diagnosis, two remain hemiparetic, and one has died. One patient could not be traced.

### MIDDLE CEREBRAL ARTERY OBSTRUCTION

There were fifteen cases of partial or complete obstruction in the middle cerebral artery.

*Case History*

A 19-year-old recently married female was found collapsed at a bus stop, where she had gone after leaving a hairdresser's shop. She was found to be hemiplegic and it was suspected that she had suffered a subarachnoid haemorrhage. Arteriography (Plate V) however showed that her middle cerebral artery was completely occluded close to its origin, the stump of the vessel being clearly seen in the right-hand picture. She slowly improved over the subsequent 2 years.

The follow-up of these fifteen patients show that only three are now well, 1 month to 3 years after diagnosis, five remain hemiparetic, and three are dead; four could not be traced. The recovery rate on the whole in this group is rather poor, which is probably to be expected as the middle cerebral artery is virtually an end-artery, and any collateral blood supply that may be able to develop would need to come through the brain substance from the small branches of the anterior and posterior cerebral arteries.

### LESIONS ELSEWHERE

Of the other patients with obstructions elsewhere, one case is worthy of note.

*Case History*

A male patient of 58 years was referred for arteriography by Dr. H. J. Crow, with the history that for 7 years he had been liable to attacks of giddiness, sometimes accompanied by a blackout, especially when he tried to run. Five years ago he had suffered a fit followed by weakness in his left arm, which had fluctuated ever since with a tendency to get worse. For 1 year he had complained of pins and needles in his left arm and leg, and on examination he had a marked thrill and bruit over the right side of his neck at the lower end. An ordinary arteriogram showed no lesion in the internal carotid artery or in the intra-cranial vessels, so that at a further session we inserted the needle the other way round and injected contrast material down the artery, producing the film shown in Plate VI. As we had no other explanation for the clinical findings, we felt that the narrowing at the lower end of the common carotid artery must be of significance. It is now eighteen months since this examination, during which time there has been no change in the patient's clinical state. He is still able to do a full-time job.

Other sites of partial obstruction in the internal carotid artery have been noted, particularly in the upper cervical region, just below the foramen lacerum. This would suggest that apart from diagnosing the condition, accurate localization should be undertaken in cases likely to come for surgical treatment.

### FOLLOW-UP

Table II shows a summary of the follow-up for the ninety-one patients. It will be noted that just over one-third of them are now well and that nearly one-quarter of them are dead, the remainder being still in some stage of disability. These figures are a little better than those given by both Marshall and Shaw (1959) and Lindgren (1958).

As far as treatment is concerned, fourteen patients had anticoagulants at one stag[illegible] or another, and of these five are now well 1 month to 3 years later, five are still hem[illegible] paretic, and four are dead. Although of course one cannot draw any conclusions fro[illegible]

TABLE II

Follow-up of 91 Cases of Arterial Obstruction

| *Result* | *Number of patients* | *Time after diagnosis* |
|---|---|---|
| Well (1 died of myocard. infarct at 2 years) | 33 | 1 month—5 years |
| Hemiparetic (15 improving, 1 worse) | 30 | 2 months—5 years |
| Dead | 20 | Up to 10 months |
| Not traceable | 8 | — |

such a small group in what was in no way a controlled trial of this form of therapy, [illegible] will be noted that those patients receiving anticoagulants did not progress very mu[illegible] better than the others.

Finally, it does appear from further analysis of these cases that the most favourab[illegible] prognosis can be given when the patient is under 50 years of age, with a slow onset [illegible] symptoms, a normal blood pressure, and a partial obstruction in the carotid arter[illegible] preferably perhaps at the upper end.

SUMMARY

In the last 5 years, ninety-one diagnoses of partial or complete obstruction in th[illegible] carotid artery or the middle cerebral artery have been made by arteriography.

A few of these cases are illustrated and an analysis has been made of the follow-u[illegible] The younger the patient and the less complete the obstruction, the better is th[illegible] prognosis.

*Acknowledgements*

My thanks are due to the many consultants who allowed me to refer to their clinic[illegible] notes and to the numerous family doctors who replied to letters of enquiry about the[illegible] patients.

REFERENCES

Hultquist, G. T. (1941). *Zeitschr. f. Kreislaufforsch.*, **33**, 657.
Lindgren, S. O. (1958). *Acta. Psychiat. Scand.* **33**, 343.
Leussenhop, A. J. (1959). *J. Neurosurgery*, **16**, 705–30.
Marshall, J. and Shaw, D. A. (1959). *Brit. Med. Journ.* **i**, 1614.
Northcroft, G. B. and Morgan, A. D. (1944). *Brit. J. Surg.* **32**, 105–107.

## Figures and Tables

**PLATE I f1:**
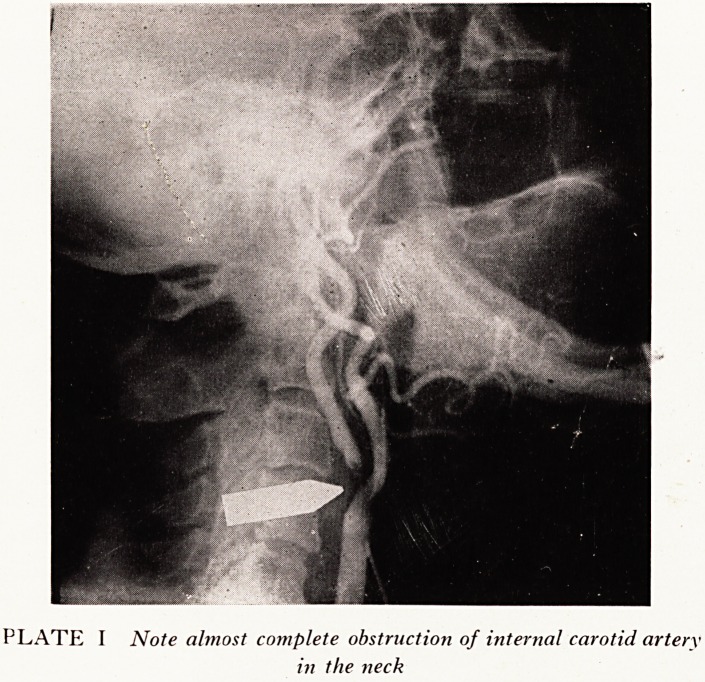


**PLATE II f2:**
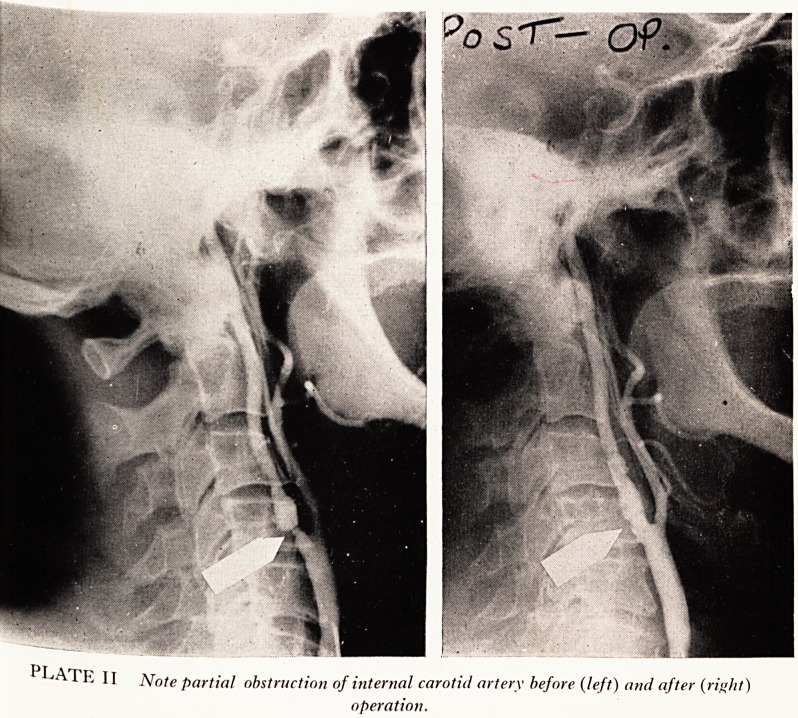


**PLATE III f3:**
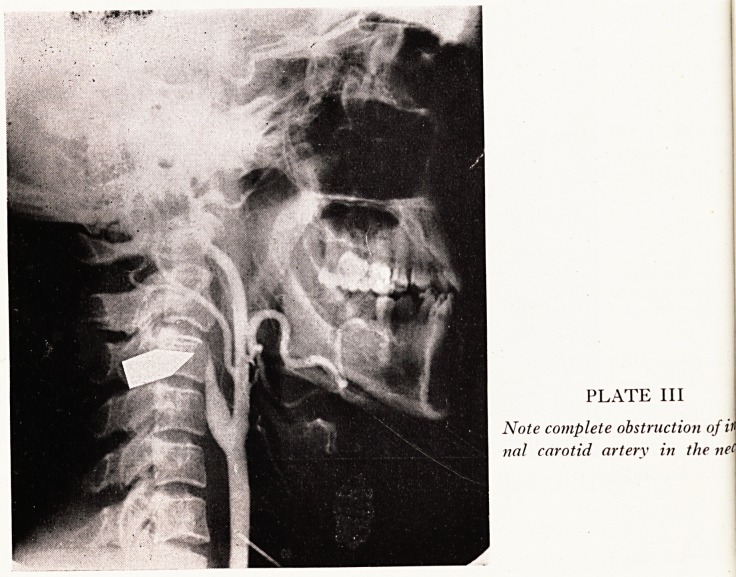


**PLATE IV f4:**
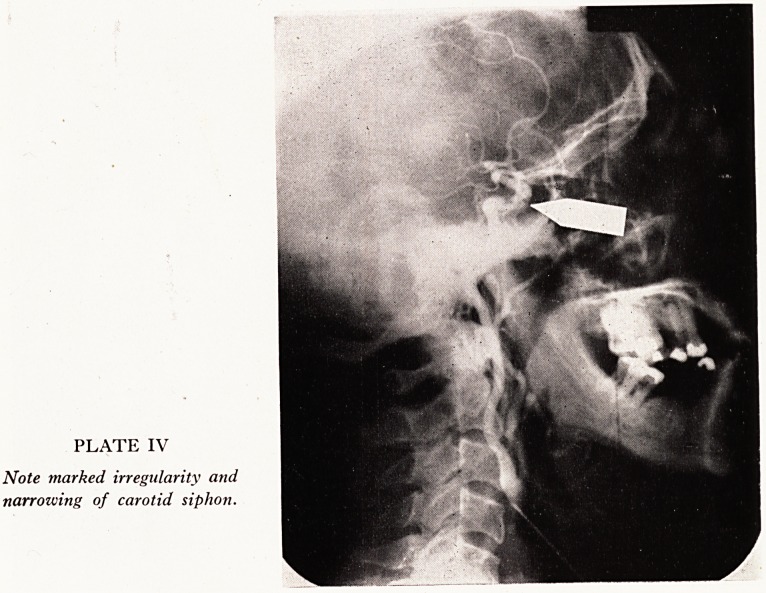


**PLATE V f5:**
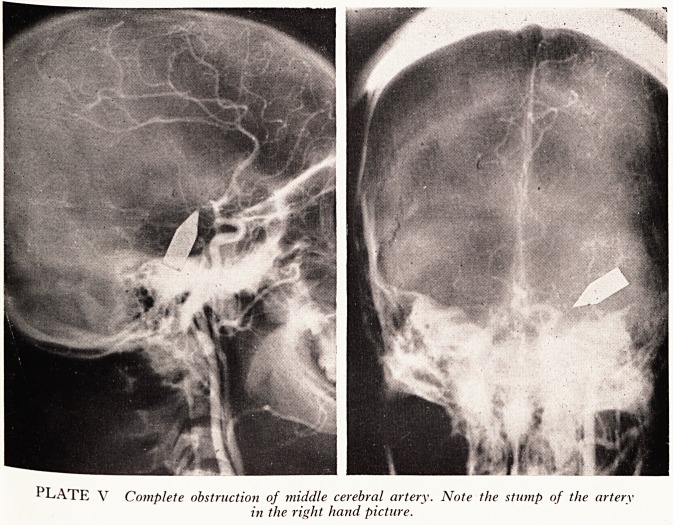


**PLATE VI f6:**